# Exploring age-related changes in saccades during cognitive tasks in healthy adults

**DOI:** 10.3389/fnbeh.2023.1301318

**Published:** 2024-01-05

**Authors:** Hee Won Yang, Jin Yeong Choe, Soo Rim Noh, Jeong Lan Kim, Ji Won Han, Ki Woong Kim

**Affiliations:** ^1^Department of Psychiatry, Chungnam National University Hospital, Daejeon, Republic of Korea; ^2^Department of Brain and Cognitive Science, Seoul National University College of Natural Sciences, Seoul, Republic of Korea; ^3^Department of Psychology, Chungnam National University, Daejeon, Republic of Korea; ^4^Department of Psychiatry, School of Medicine, Chungnam National University, Daejeon, Republic of Korea; ^5^Department of Neuropsychiatry, Seoul National University Bundang Hospital, Seongnam, Republic of Korea; ^6^Department of Psychiatry, Seoul National University, College of Medicine, Seoul, Republic of Korea

**Keywords:** saccade, non-visual task, memory, arithmetic, older adults

## Abstract

**Introduction:**

Although eye movements such as saccades are related to internal cognitive processes and are independent of visual processing, few studies have investigated whether non-visual cognitive tasks simultaneously affect horizontal and vertical saccades in younger and older adults.

**Methods:**

We recruited 28 younger adults aged 20–29 years and 26 older adults aged >60 years through advertisements in community settings. All participants were free of major psychiatric, neurological, or ocular diseases. All participants performed the mental arithmetic task (MAT) and verbal fluency task (VFT). The primary measures were saccade parameters, including frequency, mean amplitude, and mean velocity.

**Results:**

During MAT and VFT, the frequencies of horizontal and vertical saccades increased (*p* = 0.0005 for horizontal saccade in MAT; *p* < 0.0001 for horizontal saccade in VFT; *p* = 0.012 for vertical saccade in MAT; *p* = 0.001 for vertical saccade in VFT), but were comparable between MAT and VFT. The old group showed a slower vertical saccade than the young group during the tasks (*p* = 0.011 in the MAT phase; *p* = 0.006 in the VFT phase). The amplitude of the horizontal saccade decreased in both groups during MAT compared to the resting period (*p* = 0.013), but did not change significantly during VFT.

**Discussion:**

Saccade parameters can change during non-visual cognitive tasks with differences between age groups and saccade directions. This study significantly contributes to our understanding of the distinct dynamics of horizontal and vertical saccades across various age group in cognitive aging, despite its restricted focus on specific saccade parameters and cognitive tasks, and inclusion solely of cognitively normal individuals. This study highlights the importance of saccade analysis in elucidating age-related cognitive changes. In conclusion, saccades should be examined in future studies as a potential non-invasive biomarker for early detection of cognitive decline and neurodegenerative diseases.

## Introduction

As the global population continues to age, the number of people with dementia is increasing rapidly (Prince et al., 2013). There is no cure for dementia, and early diagnosis and intervention are crucial for its management (Lopez-Bastida et al., 2006); however, more than half of people with dementia do not receive a timely diagnosis. This is mainly because dementia diagnosis is largely based on documenting cognitive decline, by which time the disease has caused severe brain damage (McKhann et al., 2011). Although many biomarkers for dementia have been identified including cerebrospinal fluid, blood plasma, and anatomical and functional neuroimaging, most are either invasive, inaccurate, or expensive (Opwonya et al., 2022).

Because eye movements share many neurological and vascular substrates with cognition, early cerebral changes associated with cognitive decline may result in changes in eye movements (Holmqvist et al., 2011; Jamadar et al., 2013). Saccadic eye movements are rapid, highly stereotyped conjugate eye movements that allow humans to shift fixation from one object in the environment to the next. While there are several types of eye movements to which eye-tracking metrics can be applied, saccadic eye movements are the only type that allow humans to transmit new visual stimuli to the fovea (Kandel et al., 2013; Borys and Plechawska-Wójcik, 2017). Saccades have been extensively studied in terms of their function as part of the visual system; therefore, the association between saccades and cognition has been predominantly examined during visual tasks (Leigh and Kennard, 2004; Opwonya et al., 2022). However, saccades appear to occur not only for the purpose of visual processing, but also during thinking, and are thus referred to as “non-visual” eye movements (Ehrlichman and Micic, 2012). Saccades associated with non-visual cognitive processes have been studied extensively since initial research found increased eye movements during internal cognitive processes (Antrobus et al., 1964; Day, 1964). Although saccades may occur as a result of the need to move the eyes away from a distracting visual stimulus to minimize distraction while thinking (Glenberg et al., 1998; Phelps et al., 2006), this does not explain why people make multiple eye movements in visually impoverished settings or when the eyes are closed (Ehrlichman et al., 2007). As a result, many studies of eye movements in non-visual cognitive processes have been conducted under the premise that saccades are predominantly related to internal cognitive processes often viewed as largely independent of visual processing (Micic et al., 2010; Sousa, 2013; El Haj and Lenoble, 2018). While it is crucial to acknowledge that saccades are complex phenomena affected by both visual and cognitive factors (Leigh and Zee, 2015), these previous studies examined the modulation of saccades by internal cognitive processes in the context of non-visual cognitive tasks.

Saccades are associated with several specific cognitive processes, particularly in non-visual cognitive tasks. A high frequency of saccades is associated with activities that require retrieval from long-term memory, such as semantic or episodic memory tasks, whereas lower saccade frequencies are observed in tasks that involve working memory (Ehrlichman et al., 2007; Micic et al., 2010; Sousa, 2013; El Haj and Lenoble, 2018). Previous studies suggested that the mental arithmetic task modulates saccadic patterns in relation to cognitive load and working memory demands by requiring the manipulation and storage of numerical information, with cognitive load affecting saccade generation (Siegenthaler et al., 2014; Gao et al., 2015; Krejtz et al., 2018). The literature suggests that both verbal fluency and mental arithmetic tasks are sensitive to age-related cognitive decline. In healthy aging, a decline in verbal and numerical abilities is observed, including a decline in semantic and phonemic fluency, as well as in complex arithmetic performance. This decline is more pronounced in pathological conditions such as mild cognitive impairment and Alzheimer’s disease, where deficits in memory retrieval and arithmetic processing are early indicators of cognitive decline (Duverne and Lemaire, 2005; Sousa, 2013). The performance on these tasks not only reflect decline in specific cognitive domains, but also provides insight into broader cognitive processing resources, such as processing speed and working memory capacity, which are thought to underlie age-related cognitive changes. To our knowledge, most previous studies of saccades during non-visual saccades have been conducted exclusively in younger adults. The inclusion of older adults in this study is necessary because it allows for the examination of cognitive changes across the lifespan, providing a clearer picture of what constitutes normal versus pathological aging, and it will help to understand how cognitive processes and saccadic behavior change both as a result of normal aging and in the context of neurodegenerative diseases. Furthermore, horizontal and vertical saccades in non-visual cognitive activities have not been studied separately. Horizontal and vertical saccades are anatomically and physiologically distinct. Horizontal saccades involve an antagonistic muscle control system that is precisely coordinated by the paramedian pontine reticular formation and the abducens nucleus, allowing rapid and simultaneous eye movements in the same horizontal direction. In contrast, vertical saccades require a more complex coordination of muscle pairs orchestrated by the rostral interstitial nucleus of the medial longitudinal fasciculus and a less defined network of neural projections (Leigh and Zee, 2015). Because of this complexity, the neural control of vertical saccades is less understood, with fewer studies addressing their specific characteristics and the pathways involved (Takahashi and Shinoda, 2018). The need to study horizontal and vertical saccades separately is underscored by their different responses to aging and neurodegenerative diseases. Horizontal saccades tend to slow down with age, but it is vertical saccades that show a more pronounced susceptibility to the aging process, often presenting with greater deficits in conditions such as Parkinson’s disease and progressive supranuclear palsy. These differences suggest that each type of saccade may be uniquely affected by neural degeneration, leading to specific clinical symptoms. Thus, a separate investigation of horizontal and vertical saccades is crucial for a comprehensive understanding of their distinct neural mechanisms, their individual roles in cognitive tasks, and their potential as differential biomarkers for different neurodegenerative diseases (Takahashi and Shinoda, 2018; Irving and Lillakas, 2019).

The aim of this study was to simultaneously investigate the effects of non-visual cognitive tasks, including mental arithmetic and verbal fluency tasks, on horizontal and vertical saccades in both younger and older adults with the following hypotheses: (1) the association of saccade parameters with age group may differ between horizontal and vertical saccades; (2) the association of saccade parameters with age group may differ between types of cognitive tasks. Specifically, the frequency of saccades may increase during verbal fluency tasks compared to mental arithmetic tasks and resting period; the mean amplitude of saccades may decrease during tasks compared to resting period.

## Materials and methods

### Participants

We recruited young adults aged 20–29 years old through advertisements on the school website of Chungnam National University, and older adults aged 60 years or older through an advertisement in the bulletin of Yuseong-Gu Senior Welfare Center. Eligible participants for this study were healthy adults with no neurological or psychiatric diseases. Participants were also required to be cognitively normal, as indicated by a Mini Mental Status Examination (MMSE) score of 24 or higher (Folstein et al., 1975; Nieuwenhuis-Mark, 2010). Participants were excluded if they met any of the following exclusion criteria: (1) a history of psychiatric disorders and current treatment for psychiatric symptoms, (2) the presence of medical or neurological disorders or a history of head injury and neurological disorders, (3) hearing impairments that would interfere with the performance of auditory tasks, (4) ocular disorders or cataracts that could potentially affect pupil movement, and (5) difficulty understanding the study objectives and procedures. A total of 43 young adults were initially enrolled in the study. Of these, 15 were deemed ineligible for analysis; seven due to inadequate calibration and eight due to poor data integrity. Similarly, of the 63 older adults initially recruited, 37 were excluded from the analysis. Of these, 19 were removed due to calibration deficiencies and 18 were excluded due to poor data quality. Thus, the final analytical samples consisted of 28 young adults [17 women [60.7%] and 11 men (39.3%); mean (SD) age, 22.0 (1.6) years] and 26 older adults [13 women (50.0%) and 13 men (50.0%); mean (SD) age, 69.4 (5.9) years] as shown in Table 1. The present study protocol was reviewed and approved by the Institutional Review Board of Chungnam National University (approval number 201512-SB-034-01). Informed consent was obtained from all subjects when they were enrolled.

**Table 1:** Characteristics of the participants.

	Young (*N* = 28)	Old (*N* = 26)	Z or χ^2^	*p* ^*^
Age (years, mean ± SD)	22.0 ± 1.6	69.4 ± 5.9	−6.34	**< 0.001**
Education (years, mean ± SD)	13.7 ± 1.0	12.4 ± 3.7	−1.75	0.079
Sex (women, %)	60.7	50.0	0.63	0.429
CERAD-TS (points, mean ± SD)	87.6 ± 4.3	71.3 ± 6.9	−6.05	**< 0.001**
MMSE (points, mean ± SD)	29.8 ± 0.4	27.7 ± 1.3	−5.99	**< 0.001**
MAT (percentage of correct responses, mean ± SD)	81.6 ± 12.2	41.9 ± 16.1	−5.85	**< 0.001**
VFT (number of correct responses, mean ± SD)	19.4 ± 6.0	13.1 ± 4.7	−4.20	**< 0.001**

Abbreviations: SD, standard deviation; CERAD-TS, the total score of Consortium to Establish a Registry for Alzheimer’s Disease Neuropsychological Battery; MMSE, the total score of Mini Mental Status Examination; MAT, mental arithmetic task; VFT, verbal fluency task. *Wilcoxon-Mann–Whitney tests for continuous variables and chi square tests for categorical variables. The bold values mean statistically significant.

### Procedures

Each participant was asked to sit comfortably on a height-adjustable chair fixed to the floor, and to fit his or her chin on a chinrest fixed to the desk (Oguro et al., 2004), in a soundproof room previously used for EEG studies to ensure a stable and distraction-free environment. Before the main experiments, all participants performed practice trials to ensure that they understood the tasks. Although the exact decibel level was not quantified, the audibility of the voice commands recorded by a professional voice actor was checked to ensure that they were clear to all participants before the main experiment began, thus ensuring the reliability of the auditory presentation.

We recorded participants’ resting eye movements for 10 s while they watched a blank screen before the cognitive tasks. We then asked participants to watch the screen while performing the cognitive tasks and recorded their eye movements. Each participant performed two cognitive tasks, the mental arithmetic task (MAT) and the verbal fluency task (VFT), which were presented in random order. In the VFT, we asked participants to generate as many words as possible in the animal or vegetable category within 60 s. In the MAT, we asked participants to click the mouse button after completing the mental calculation, to provide a verbal answer to each arithmetic question presented audibly, and to click a mouse button again to proceed to the next question. The MAT consisted of six easy, six medium, and six difficult multiplication problems (Ahern and Beatty, 1979). Each multiplication problem was presented for two seconds, and the participants were asked to answer each problem within 2 min. These multiplication problems were presented randomly in one of eight list types. In each sequence, the same difficulty level did not occur more than three times in a row. At the easy level, the multiplicands ranged from three to nine, and the multipliers ranged from 13 to 19. At the medium level, the multiplicands ranged from 6 to 14 and the multipliers ranged from 13 to 27. At the difficult level, the multiplicands ranged from 11 to 19 and the multipliers ranged from 16 to 27. The easy, medium, and difficult level problems took the form [3, 4, 5, 6, 7, 8, 9] x [13, 14, 15, 16, 17, 18, 19], [6, 7, 8, 9, 10, 11, 12, 13, 14] x [13, 14, 15, 16, 17, 18, 19, 20, 21, 22, 23, 24, 25, 26, 27], and [11, 12, 13, 14, 15, 16, 17, 18, 19] x [16, 17, 18, 19, 20, 21, 22, 23, 24, 25, 26, 27], respectively. We then excluded items where the result of the multiplication automatically comes out when it’s a multiple of 10, such as 10 × 15, because they were easy to remember from the multiplication table. Recognizing the challenges of auditory differentiation in the elderly, particularly in distinguishing between the Korean numbers 1 and 2, the language pathologists checked and excluded items that presented an overlap of these numbers, such as 21×22 and 12×11, to ensure clarity and avoid confusion. In addition, we created four practice problems in the same way, consisting of two levels of difficulty and always presented before the experimental multiplication problems.

### Eye tracking

Eye movements were recorded using a Tobii Pro X3-120 Eye Tracker (Tobii Technology, Danderyd, Sweden), which tracks eye movements using a corneal reflection screen with a combination of dark and light pupils (Tobiipro, 2018). We performed a 5-point calibration of the eye tracker when both eyeballs were detected by the tracker before beginning the experiments. The sampling rate, accuracy, and precision of the tracker were 120 Hz, 0.4°, and 0.24°, respectively (Tobiipro, 2023). We controlled the experimental procedures using Tobii Studio 3.4.4 software (Tobii Technology, Danderyd, Sweden) on a Dell PRECISION workstation. We projected the workstation monitor onto a screen with a resolution of 1,024 × 768 pixels (200 cm × 152 cm) at a distance of approximately 232 cm from the participant. This ensured accurate capture of natural eye movements and extended the measurement range, as the standard computer monitor often limits the measurable range during non-visual cognitive tasks, as found in our pilot study.

### Cognitive assessments

A trained neuropsychologist administered the Korean version of the Consortium to Establish a Registry for Alzheimer’s Disease neuropsychological assessment battery (CERAD-NP) (Lee et al., 2002, 2004). The cognitive function of each participant was evaluated using the total score of Consortium to Establish a Registry for Alzheimer’s Disease Neuropsychological Battery (CERAD-TS) and the total score of the MMSE (Seo et al., 2010).

### Statistical analysis

We analyzed saccades during 60-s response period for VFT. For MAT, saccades were analyzed during the calculation period, which is defined as the time from the presentation of each problem to the participant’s mouse click before their response. We analyzed the eye movement tracking data using R software version 3.3.2 (RR Core Team, 2017). First, we smoothed the raw data using a 3-point moving average filter to reduce noise and improve saccade detection. We then defined eye movements with a velocity of 100°/s or more as saccades to reduce the false-positive rate of saccades (Dowiasch et al., 2015). We counted each saccade as an independent saccade only if two saccades occurred at an interval of 50 ms or longer or were separated by at least six consecutive data points slower than 100°/s (Amit et al., 2017; Abeles et al., 2020). We measured the frequency (numbers/s), mean amplitude (°), and mean velocity (°/s) of horizontal and vertical saccades in the resting and task periods. For each saccade, the horizontal and vertical components were measured separately on a two-dimensional plane.

To compare demographic and clinical characteristics and saccade parameters between age groups, we performed Pearson’s chi-square test for categorical variables and the non-parametric Wilcoxon-Mann–Whitney test for continuous variables due to their non-normal distribution. We examined the effects of age group and task on the frequency, mean amplitude, and mean velocity of saccades using two-way repeated measures analysis of variance (rmANOVA) because our research design involved repeated observations under different task conditions and across two different age groups. Prior to rmANOVA, we performed Shapiro–Wilk tests to confirm the normality assumption. We also evaluated the skew and kurtosis values to gain a more detailed understanding of the distribution of all variables used in rmANOVA (Mishra et al., 2019). We transformed the frequency, mean amplitude and mean velocity of saccades into ranks using the Aligned Rank Transform (ART) because they were not normally distributed (Wobbrock et al., 2011). In the rmANOVA, we classified age groups (younger and older) as a between-subjects variable and the periods (resting, VFT, and MAT) as a within-subjects variable. We performed pairwise comparisons between tasks using Tukey’s method, which was selected because of its strict control of the familywise error rate in the context of multiple testing and its high statistical power, ideal for the balanced design of our study. *p* < 0.05 was considered statistically significant. We calculated effect size using partial omega squared (ω^2^), providing an estimate of how much variance in the dependent variable are accounted for by the explanatory variables. Partial ω^2^ is widely viewed as a lesser biased measure of effect size, especially when sample sizes are small. A larger partial ω^2^ value would indicate a larger contribution of the factor to the observed variance in saccade parameters, suggesting a stronger effect of age group or task type on saccades (Olejnik and Algina, 2003). ART and rmANOVA were performed using the R package ARTool (Kay and Wobbrock, 2018). All other statistical analyses were performed using IBM SPSS Statistics for Widows, Version 22.0 (IBM Corp, Released 2013, Armonk, NY).

## Results

The old group showed lower CERAD-TS and MMSE scores than the young group. The old group also performed worse in the MAT and VFT than the young group. However, the level of education and the proportion of women were comparable between the two groups (shown in Table 1).

During MAT and VFT, the frequency of both horizontal and vertical saccades increased. However, the changes in mean amplitude and mean velocity differed between horizontal and vertical saccades. The mean amplitude of the horizontal saccade decreased, while the velocity of the horizontal saccade remained stable. In contrast, the mean amplitude of the vertical saccade decreased, while the mean velocity of the vertical saccade increased (shown in Table 2).

**Table 2:** Changes in the saccadic eye movements associated with non-visual cognitive tasks.

		Resting	Mental arithmetic task	Verbal fluency task								
	Young	Old	*Z*	*p* ^*^	Young	Old	*Z*	*p* ^*^	Young	Old	*Z*	*p* ^*^
**Frequency (n/s)**												
Horizontal	0.8 ± 0.8	0.8 ± 0.7	−0.39	0.696	1.6 ± 1.3	1.5 ± 1.5	−0.92	0.359	1.8 ± 2.1	1.7 ± 1.2	−0.04	0.972
Vertical	1.9 ± 1.5	1.4 ± 1.2	−0.98	0.328	2.9 ± 2.5	2.3 ± 2.2	−0.99	0.324	2.9 ± 2.8	2.5 ± 1.7	−0.47	0.640
**Mean amplitude (°)**												
Horizontal	3.9 ± 3.1	3.4 ± 2.1	−0.10	0.921	2.3 ± 1.2	2.3 ± 0.6	−0.74	0.457	3.1 ± 1.6	3.1 ± 1.1	−0.73	0.467
Vertical	2.3 ± 0.9	2.9 ± 1.6	−0.96	0.336	2.4 ± 0.7	2.3 ± 0.6	−0.69	0.489	2.5 ± 0.5	2.4 ± 0.7	−0.59	0.556
**Mean velocity (°/s)**												
Horizontal	171.4 ± 44.9	168.1 ± 38.0	−0.01	0.991	168.5 ± 34.6	168.5 ± 38.1	−0.02	0.986	172.2 ± 30.4	169.1 ± 25.0	−0.14	0.890
Vertical	174.2 ± 48.7	164.2 ± 42.2	−0.94	0.346	194.2 ± 47.6	161.8 ± 25.5	−2.77	**0.006**	186.6 ± 41.9	159.8 ± 18.0	−2.94	**0.003**

All values are presented as mean ± standard deviation. *Comparisons between young and old groups using Wilcoxon-Mann–Whitney tests. The bold values mean statistically significant.

**Figure 1:**
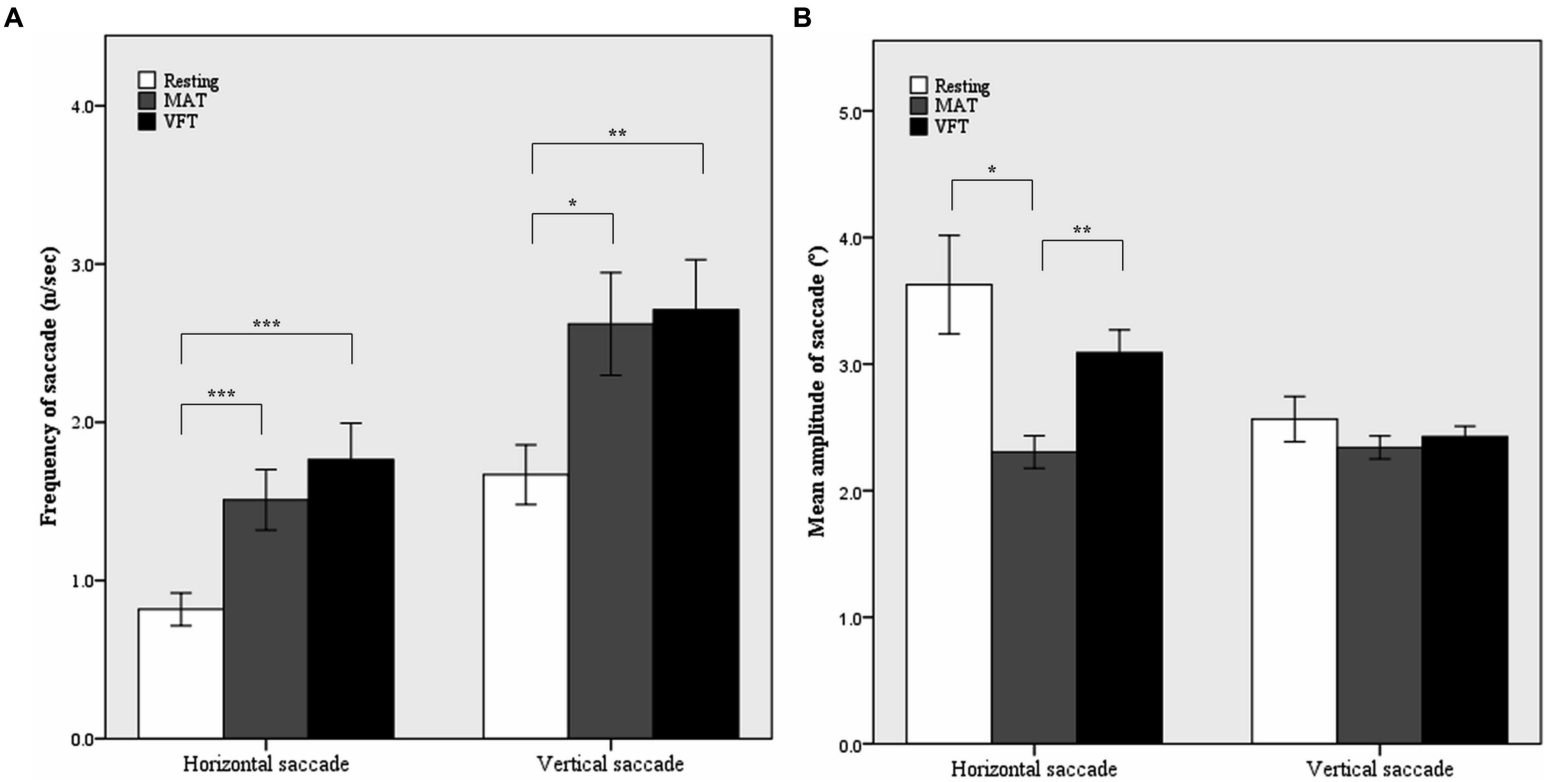
Mean frequencies **(A)** and mean amplitudes **(B)** of horizontal and vertical saccades in the resting and non-visual cognitive periods. MAT, mental arithmetic task; VFT, verbal fluency task. Error bars represent standard error. ^*^p < 0.05, ^**^p < 0.01, ^***^p < 0.001 by Tukey’s *post hoc* comparison.

In the two-way rmANOVA (shown in Table 3), the main effect of task was significant for the frequency of horizontal [*F* (2, 104) = 15.68, *p* < 0.001, ω^2^ = 0.22] and vertical [*F* (2, 104) = 7.35, *p* = 0.001, ω^2^ = 0.11] saccades. During the tasks, the frequency of horizontal saccades almost doubled and that of vertical saccades increased by approximately 60%. In *post hoc* comparisons, the frequencies of both horizontal and vertical saccades were significantly higher in MAT and VFT than in the rest (*p* = 0.0005 for horizontal saccade in MAT; *p* < 0.0001 for horizontal saccade in VFT; *p* = 0.012 for vertical saccade in the MAT; p = 0.001 for vertical saccade in VFT), but were comparable between the MAT and VFT (*p* = 0.270 for horizontal saccade; *p* = 0.763 for vertical saccade, shown in Figure 1A).

**Table 3:** Effects of age and tasks on the saccadic eye movements.

	Age group	Tasks	Age group*Tasks							
	*F* ^*^	*p* ^*^	ω^2^	*F* ^*^	*p* ^*^	ω^2^	*Posthoc* ^**^	*F* ^*^	*p* ^*^	ω^2^
**Frequency (n/sec)**										
Horizontal	0.10	0.758	0.00	15.68	**< 0.001**	0.22	M, V > R	0.44	0.646	0.00
Vertical	0.45	0.505	0.01	7.35	**0.001**	0.11	M, V > R	1.27	0.285	0.01
**Mean amplitude (°)**										
Horizontal	0.00	0.978	0.00	7.34	**0.001**	0.11	V, R > M	0.48	0.620	0.00
Vertical	0.08	0.775	0.00	0.53	0.592	0.00	–	1.54	0.216	0.01
**Mean velocity (°/sec)**										
Horizontal	0.01	0.909	0.00	0.76	0.472	0.02	–	0.01	0.988	0.00
Vertical	9.69	**0.003**	0.14	2.31	0.104	0.02	–	0.72	0.490	0.00

M, mental arithmetic task; V, verbal fluency task; R, resting. ^*^Two-way repeated measures analysis of variance. Effect size was expressed as partial ω^2^. ^**^p value was adjusted using Tukey’s method. The bold values mean statistically significant.

The main effect of task was also significant for the mean amplitude of the horizontal saccade (F(2, 104) = 7.34, *p* = 0.001, ω^2^ = 0.11). As shown in Table 2 and Figure 1B, the mean amplitude of the horizontal saccade decreased by approximately 40% in the young group and by approximately 30% in the old group during the MAT compared to the resting period (*p* = 0.013). However, it did not change significantly during VFT (*p* = 0.857).

**Figure 2:**
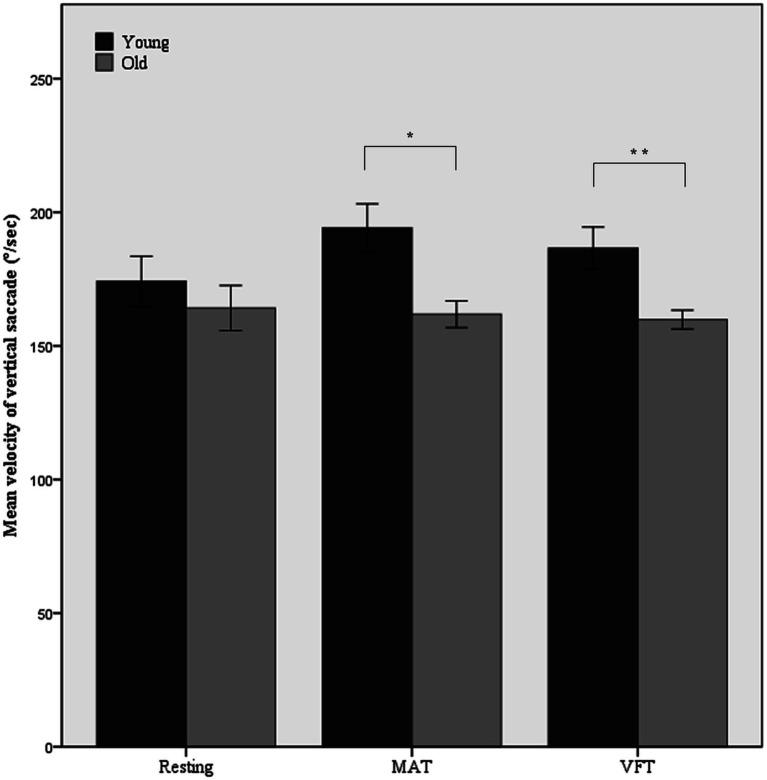
Mean velocity of vertical saccade in the resting and non-visual cognitive task periods. MAT, mental arithmetic task; VFT, verbal fluency task. Error bars represent standard error. ^*^p < 0.05, ^**^p < 0.01.

The main effect of age on the mean velocity of vertical saccades was significant (*F*(1,52) = 9.69, *p* = 0.003, ω^2^ = 0.14). During the tasks, the old group showed slower vertical saccades than the young group. As shown in Figure 2, the difference in the mean velocity of vertical saccades between the young and old groups increased during MAT and VFT compared with the resting period (mean difference = 10.0, *p* = 0.346 in the rest; mean difference = 29.4, *p* = 0.011 in MAT; mean difference = 22.3, *p* = 0.006 in VFT). A task-associated increase in the mean velocity of the vertical saccade was not observed in the old group.

No interactive effects between age group and task were found for any of the saccade parameters (shown in Table 3).

## Discussion

This study was the first to demonstrate that the frequency of horizontal and vertical saccades increased during MAT to the same extent as during VFT. Although many previous studies have reported an increase in the frequency of saccades during long-term memory tasks, such as verbal fluency and episodic recall tasks (Ehrlichman et al., 2007), the association of saccades with arithmetic tasks has never been investigated.

In previous studies, the increase in the frequency of saccades during long-term memory tasks was greater than that during working memory and executive function tasks (Ehrlichman et al., 2007; Micic et al., 2010; Sousa, 2013; El Haj and Lenoble, 2018). The mechanism underlying the increase in saccades during long-term memory tasks is not fully understood. In the hypothetical model proposed by Ehrlichman and colleagues (Ehrlichman et al., 2007), the superior colliculus plays a key role as a saccade generator by interacting with related brain regions. Saccades are more frequent during high-retrieval tasks because activation of the medial temporal lobe for retrieval of long-term memory leads to inhibition of the striatum and consequent disinhibition of saccadic neurons in the superior colliculus. Arithmetic tasks may also increase the frequency of saccades by activating bilateral posterior superior parietal lobules which are known to be associated with horizontal saccades (Knops et al., 2009; Zhu et al., 2019). Knops et al. previously proposed that number processing recruits a brain area that is tightly interconnected with the nearby posterior superior parietal lobule, which is involved in saccadic and attentional control (Knops et al., 2009). However, the causal role of the posterior superior parietal lobule in arithmetic has not yet been established.

The frequency of vertical and horizontal saccades increased in both MAT and VFT. However, the mean amplitude of the horizontal saccade decreased only in the MAT. This may be due, at least in part, to the difference in cognitive load between MAT and VFT. The amplitude of saccades has also been reported as a potential measure of cognitive workload; and a previous study has shown that amplitude decreases as the difficulty of the working memory task increases (May et al., 1990). However, no studies have yet directly investigated the association between the amplitude of saccades and the difficulty of arithmetic tasks. However, several previous studies have shown that the amplitude of the microsaccades increased as the difficulty of the arithmetic task increased (Siegenthaler et al., 2014; Krejtz et al., 2018). Microsaccades are eye movements that occur during an attempt at visual fixation, and their amplitudes tend to change in the opposite direction of a saccade (Siegenthaler et al., 2014; Gao et al., 2015; Krejtz et al., 2018). In addition, the mean amplitude of the vertical saccade did not change significantly during MAT in the present study. Horizontal and vertical saccades are generated independently by different groups of burst neurons: horizontal saccades by excitatory burst neurons located in the paramedian pontine reticular formation, and vertical saccades by excitatory burst neurons located in the paramedian pontine reticular formation and the rostral interstitial nucleus of the medial longitudinal fasciculus (Leigh and Zee, 2015). Vertical burst neurons are known to be more sparsely scattered than horizontal ones (Buttner-Ennever, 2008), and their amplitudes are determined by the total number of burst neuron spikes (Moschovakis and Highstein, 1994).

To the best of our knowledge, this is the first study to reveal the associations between horizontal and vertical saccades and non-visual cognitive tasks in both younger and older adults. All previous studies investigated only horizontal saccades or all saccades without distinguishing between horizontal and vertical saccades (May et al., 1990; Ehrlichman et al., 2007; Ryabchikova et al., 2009; Micic et al., 2010; Sousa, 2013; El Haj and Lenoble, 2018; Walsh, 2021). In the present study, changes in mean amplitude associated with MAT showed discrepancies between horizontal and vertical saccades, and changes in mean velocity associated with age also differed between horizontal and vertical saccades. A decrease in mean amplitude during MAT was found only for horizontal saccades, whereas an increase in mean velocity during MAT and VFT in younger adults was found only for vertical saccades. These results suggest that horizontal and vertical saccades should be investigated separately in future studies.

This study also found that older adults showed slower vertical saccades during MAT and VFT than younger adults. Although the age-associated change in the velocity of saccades during visual cognitive tasks or resting states has been repeatedly reported (Huaman and Sharpe, 1993; Munoz et al., 1998; Irving et al., 2006; Yang and Kapoula, 2006; Bonnet et al., 2013; Irving and Lillakas, 2019), it has never been investigated during non-visual cognitive tasks. Although there were no differences between specific cognitive tasks, a trend toward greater differences in the velocity of saccades was observed between younger and older adults during task performance compared to rest. This suggests that age-related changes in the velocity of saccades may be related to factors other than specific cognitive domains. Older adults with age-associated cognitive decline may not have sufficient resources to meet the cognitive demands of difficult tasks (Treitz et al., 2007; Cansino, 2009). As a result, cognitive burden and eye movement changes induced by cognitive tasks of the same difficulty may differ between older and younger adults. In addition, eye movement is a physical function produced by the coordinated efforts of extraocular muscles and related motor neurons (Leigh and Zee, 2015). The excitability of motor neurons and general motor speed decrease with age (Rossini et al., 1992; Salthouse, 2000). These physical changes may lead to changes in saccade parameters in the elderly. However, it remains unclear whether age-related changes may occur in other cognitive domains or tasks, as this study included limited cognitive tasks such as MAT and VFT. Therefore, changes in saccade parameters across tasks at different points in the life span, especially between younger and older adults, need to be investigated in future studies.

In contrast to mean velocity, the frequency of saccades was comparable between older and younger adults in our study. The decline in saccade frequency may be due to the general motor slowing and reduced excitability of motor neurons evident during aging (Rossini et al., 1992; Salthouse, 2000), whereas saccadic velocity is determined by the spike density of saccadic burst neurons (King and Fuchs, 1979; Missal et al., 2000). A previous study reported that, on average, older adults aged 88.3 years had a lower frequency of saccades than younger adults aged 19.9 years (Sousa, 2013). However, another study found that the frequency of saccades was comparable between older adults aged 69 years and younger adults aged 22 years, on average (Walsh, 2021). In the current study, the older adults aged 69.4 years showed a lower frequency of vertical saccades in both resting and cognitive task periods than younger adults aged 22.0 years on average, but the differences between them were not statistically significant. Overall, these results suggest that the frequency of vertical saccades may also decrease with age, but becomes significant in older adults aged 80 years or older.

This study had several limitations. First, this study included only cognitively normal subjects, although the changes in saccades in subjects with cognitive disorders may differ from those in subjects with normal cognition. Second, this study could not directly analyze microsaccades because of the limited resolution of the eye tracker. Third, this study did not examine the direction of horizontal and vertical saccades, although the the brain regions, neural circuits, and extraocular muscles involved in saccades may differ depending on the direction of saccades. Fourth, we did not measure the peak velocity of saccades. However, mean velocity is an acceptable measure of saccadic velocity for relatively small saccades with a symmetrical velocity waveform (Di Stasi et al., 2013). Fifth, the time periods for measuring saccades in the three different conditions were very different, which could lead to biases in measures of eye movements related to time. However, in contrast to peak velocity, saccade duration and mean velocity were insensitive to increasing time-on-task (Di Stasi et al., 2010, 2013). Sixth, some of the potentially confounding factors, such as general motor speed, performance anxiety, and cognitive burden associated with the tasks. Finally, the number of participants was small and thus subject to limited statistical power.

In conclusion, this study demonstrated that saccade parameters can change during non-visual cognitive tasks and that these changes in saccade parameters may differ between younger and older adults. In addition, age- and task-associated changes in saccades may differ between horizontal and vertical saccades. Assessing saccade changes during non-visual cognitive tasks may help to identify subtle brain changes due to aging and neurodegenerative diseases. In future research, saccades should be examined as a potential non-invasive biomarker for cognitive disorders.

## Data availability statement

The raw data supporting the conclusions of this article will be made available by the authors, without undue reservation.

## Ethics statement

The studies involving humans were approved by Chungnam National University Institutional Review Board. The studies were conducted in accordance with the local legislation and institutional requirements. The participants provided their written informed consent to participate in this study.

## Author contributions

HY: Formal analysis, Investigation, Validation, Visualization, Writing – original draft, Writing – review & editing. JC: Conceptualization, Formal analysis, Methodology, Writing – original draft. SN: Conceptualization, Funding acquisition, Methodology, Project administration, Supervision, Writing – review & editing. JK: Resources, Supervision, Writing – review & editing. JH: Formal analysis, Investigation, Supervision, Validation, Writing – review & editing. KK: Conceptualization, Methodology, Project administration, Supervision, Writing – review & editing.
